# An atypical ALS with PSP-like symptoms caused by *ANXA11* p.D40G mutation: A case report and literature review

**DOI:** 10.3389/fneur.2023.1086264

**Published:** 2023-02-16

**Authors:** Xin Zhang, Juan Gao, Chunling Chi, Zhenzhen Zhao, Piu Chan, Jinghong Ma

**Affiliations:** ^1^Department of Neurology, Xuanwu Hospital of Capital Medical University, Beijing, China; ^2^Department of Neurology, Baoding No.1 Central Hospital, Baoding, China; ^3^Department of Neurology, The Fourth Affiliated Hospital of Harbin Medical University, Harbin, China; ^4^Department of Geriatrics Center, Shenyang No.4 People's Hospital of China Medical University, Shenyang, China

**Keywords:** amyotrophic lateral sclerosis, ANXA11, genotype, phenotype, progressive supranuclear palsy

## Abstract

**Background:**

*ANXA11* mutations were first reported to be associated with amyotrophic lateral sclerosis (ALS) in 2017. Several studies have investigated the prevalence of *ANXA11* mutations in different populations, while less is known about the spectrum of phenotypes and the genotype–phenotype correlation with this gene mutation.

**Case presentation:**

Here, we report a 74-year-old man who was initially diagnosed with progressive supranuclear palsy (PSP) because of repeated falls, slight upward gaze palsy, and mild cognitive dysfunction at the onset. He finally turned out to be ALS with more and more prominent limb weakness and atrophy, together with the evidence of chronic neurogenic change and ongoing denervation on electromyography. Brain magnetic resonance imaging showed extensive cortical atrophy. A missense mutation c.119A > G (p.D40G) on the *ANXA11* gene was identified using whole-exome sequencing, which confirmed the diagnosis of ALS. We performed a systematic review of the literature about ALS-relevant cases with *ANXA11* mutations and identified 68 affected subjects and 29 variants with the *ANXA11* gene. We summarized the phenotypes of *ANXA11* mutations and the clinical characteristics of nine patients harboring the *ANXA11* p.D40G variant including our case.

**Conclusions:**

The phenotype of *ANXA11*-related cases is heterogeneous, and most cases showed typical ALS, while some could also have the characteristics of frontotemporal dementia (FTD) and PSP, even inclusion body myopathies (hIBM) occurred in familial ALS (FALS). Our patient presented with ALS with a co-morbid PSP-like symptom (ALS-PSP) phenotype, which has not been reported. Except for our patient, the remaining eight patients with the *ANXA11* p.D40G variant presented with a classical ALS phenotype without cognitive impairment.

## Introduction

Amyotrophic lateral sclerosis (ALS) is a malignant neurodegenerative disorder with a substantial heritable component. About 60% of familial ALS (FALS) and 10% of sporadic ALS (SALS) have genetic variations (1, 2). To date, more than 30 genes have been reported to be associated with ALS (3). In European populations, the most common mutation was the *C9orf72*, followed by *SOD1*, while in Asians, the most common mutation was the *SOD1* (4). Until 2017, *ANXA11* mutations were first reported to be associated with ALS (5). There were a few reports about *ANXA11* variation, and most of the reported cases presented as typical ALS phenotype, and some with frontotemporal dementia (FTD). Herein, we report a case of atypical ALS with *ANXA11* gene mutation who first showed obvious extrapyramidal symptoms and repeated falls and was initially misdiagnosed as progressive supranuclear palsy (PSP). We performed a systematic review of the literature to investigate the genotype–phenotype correlation of *ANXA11* and the clinical phenotypes with the *ANXA11* p.D40G mutation.

## Case presentation

**Figure 1:**
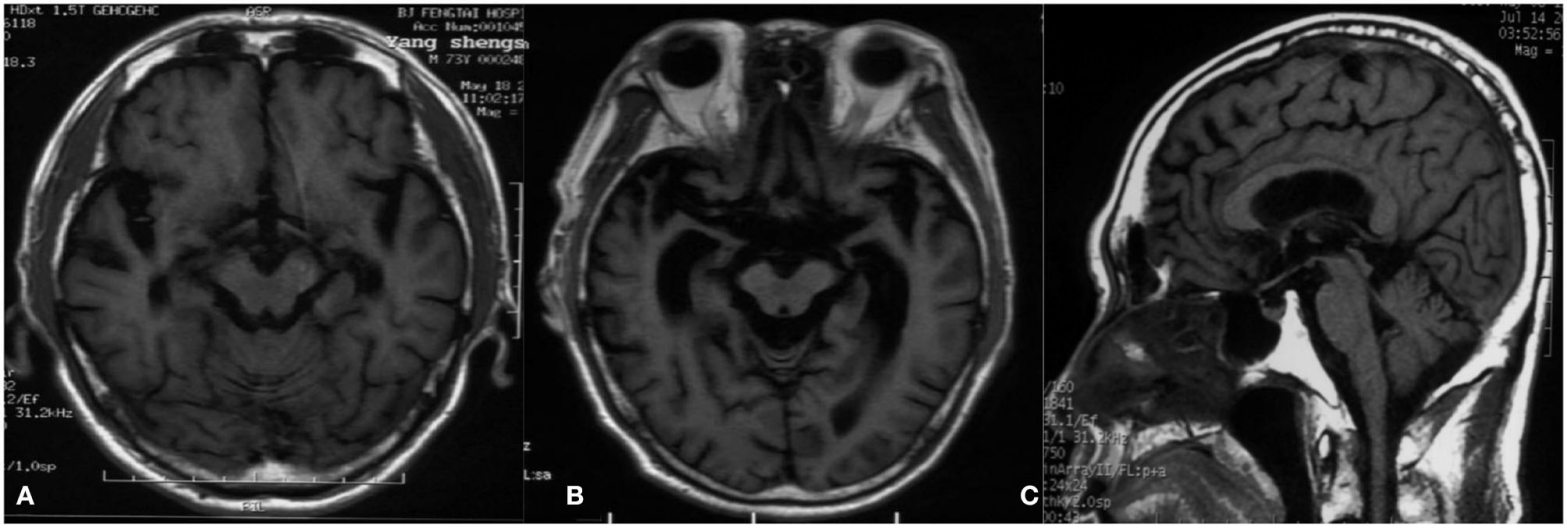
Magnetic resonance imaging. **(A)** T1-weighted image 2 months after the onset of the disease shows extensive cortical atrophy. **(B)** T1-weighted image 12 months after the onset of the disease reveals severe cortical atrophy, and the degree of atrophy gradually increased compared to A. **(C)** T1-weighted image 4 months after the onset of the disease demonstrates that the midbrain volume was comparatively well preserved compared with cortical atrophy.

**Figure 2:**
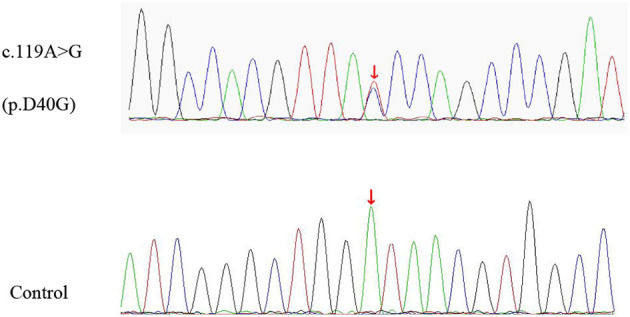
The missense variant of *ANXA11* identified in our patient. Sequence chromatograms of polymerase chain reaction (PCR) show the heterozygous c.119A > G (p.D40G) variant **(upper lane)** compared with healthy control **(lower lane)**.

A 74-year-old man presented to our clinic due to unsteady walking and repeated falls for 13 months, accompanied by a slightly drooped head and bradykinesia. At the same time, his daughter noticed that he would occasionally have forced laughter. Twelve months ago, he developed slurred speech and weakness in bilateral arms. Neurologic examinations revealed mild cognitive dysfunction (the Mini-Mental State Examination score was 23/30 and the Montreal Cognitive Assessment score was 22/30, with 11 years of education), dysarthria, slight upward gaze palsy, the muscle strength of bilateral arms was grade 5^−^/5, bradykinesia, slightly increased muscle tone in limbs except for the neck, postural instability, hyperreflexia, and positive Babinski signs bilaterally. Brain magnetic resonance imaging showed extensive cortical atrophy (Figure 1). Electromyography showed no abnormality. Within the next 9 months, his symptoms worsened rapidly, and he became wheelchair-bound and developed significantly slurred speech until mutism. At that time, physical examination showed a drooped head, mutism, muscle strength of extremities was grade 0, hypermyotonia, hyperreflexia, positive Babinski signs bilaterally, carpopedal contracture, and fasciculation in hands. Electromyography showed chronic neurogenic change and ongoing denervation. A missense mutation c.119A > G (p.D40G) of the *ANXA11* gene was identified using whole-exome sequencing and verified by Sanger sequencing (Figure 2). In the early stages of the disease, our patient showed postural instability and slight supranuclear gaze palsy and was misdiagnosed as suggestive PSP in one of the best neurological hospitals in Beijing, later because of a gradual decline in cognitive function, FTD was also suggested by an experienced neurologist. As the disease progressed rapidly, he finally turned out to be ALS. Apart from the chronic obstructive pulmonary disease, his medical history was unremarkable. He denied a family history of ALS and related disorders. He is a retired worker living in Beijing for a long time, with no history of special chemical exposure. He was initially treated with levodopa (375 mg/day) and the dosage gradually increased to 750 mg/day but without significant improvement except that muscle rigidity improved to some extent. At the 6-month follow-up, which was 19 months after the onset of the disease, he was bedridden and had a tracheostomy because of repeated pneumonia.

## Review of ALS-relevant cases with *ANXA11* mutations

**Figure 3:**
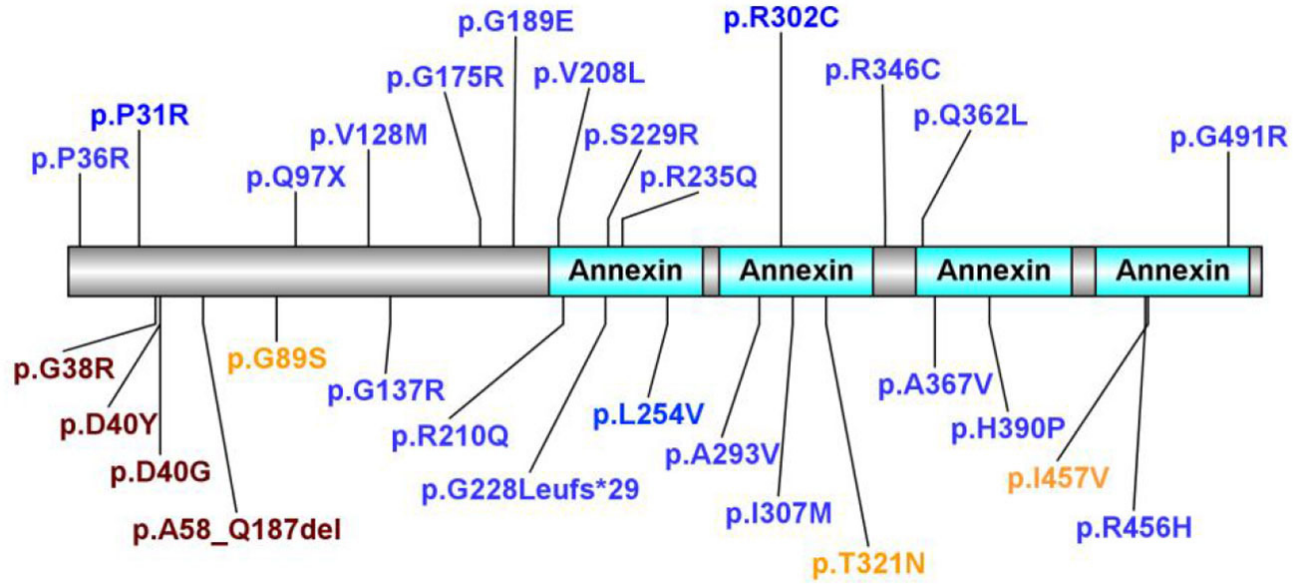
In total, 29 *ANXA11* variants identified. In total, four variants considered to be pathogenic or likely pathogenic are marked in purple. Variants considered to be benign or likely benign are marked in orange. Variants of uncertain significance are marked in blue.

A literature review was performed by searching PubMed and China National Knowledge Infrastructure (CNKI) (from their inception until February 2022) using the keywords: “annexin A11,” “*ANXA11*,” “ALS,” and “amyotrophic lateral sclerosis.” Relevant articles describing any ALS-relevant case with *ANXA11* mutations were selected. Nine articles were related to studies of interest. We identified 68 ALS-relevant cases with *ANXA11* mutations including our case, and the phenotypes of each case are listed in Table 1 (5–13). As shown in Supplementary Figure S1, they came from China (32.35%, 22/68), Brazil (19.12%, 13/68), Korea (19.12%, 13/68), multicenter (including the United States, the UK, Italy, Spain, Germany, Ireland, Canada, the Netherlands, Belgium, and New Zealand, 17.65%, 12/68), and France (11.76%, 8/68). According to the previously reported cases, most (80.88%, 55/68) of the *ANXA11* variants presented with typical ALS phenotype, and one (1.47%, 1/68) showed ALS with co-morbid FTD (ALS-FTD), while our patient (1.47%, 1/68) showed ALS with co-morbid PSP-like symptoms (ALS-PSP), which has not been reported. The phenotypes of *ANXA11* mutations are summarized in Supplementary Figure S2. We identified 29 *ANXA11* variants marked in Figure 3, and four variants (13.79%, 4/29) are considered to be pathogenic or likely pathogenic, including p.D40Y, p.D40G, p.G38R, and p.A58_Q187del (7–9, 11). We identified nine patients harboring the *ANXA11* p.D40G variant including our case. As shown in Supplementary Table S1, except for our patients, they all presented with classical ALS phenotypes without cognitive impairment (5, 9, 11). The onset age of six patients (66.67%, 6/9) exceeded 70 years of age, and the initial symptoms of six patients (66.67%, 6/9) were bulbar dysfunction.

**Table 1:** Clinical phenotypes of 68 patients with *ANXA11* mutations.

**References**	**Source**	**Study patients**	**Numbers (%) with *ANXA11* mutation**	**Phenotype**	**Characteristic**	**Age of onset (years or median years)**	**Initial symptoms**
Nunes Gonçalves et al. (6)	Brazil	107 FALS	2 (1.87%)	FALS	Rapidly progressive	NA	NA
Leoni et al. (7)	Brazil	11 cases from 3 Brazilian families	9 (81.82%)	hIBM	Slowly progressive	NA	9 limb
			1 (9.09%)	FALS	Rapidly progressive; typical	NA	1 limb
			1 (9.09%)	hIBM + ALS	Rapidly progressive; typical	NA	1 limb
Smith et al. (5)	Multicenter^*^	694 FALS	9 (1.30%)	FALS	Typical	(70)	4 bulbar + 4 limb + 1 mixed
	Britain	180 SALS	3 (1.67%)	SALS	Typical	53, 65, 72	2 bulbar + 1 limb
Teyssou et al. (8)	France	150 FALS	6 (4%)	FALS	NA	(68.5)	3 bulbar + 3 limb
		180 SALS	2 (1.11%)	SALS	NA	33, 61	2 limb
Nahm et al. (9)	Korea	500 SALS	13 (2.60%)	SALS	NA	(56)	7 bulbar + 6 limb
Tsai et al. (10)	Taiwan	42 FALS +244 SALS	8 (2.80%)	1 SALS	Rapidly progressive	61	1 bulbar
				7 ALS^*^	NA	NA	NA
Zhang et al. (11)	China	353 SALS	8 (2.27%)	SALS	NA	(57)	2 bulbar + 6 limb
		18 FALS	1 (5.56%)	FALS	NA	71	1 bulbar
		12 ALS-FTD	1 (8.33%)	ALS-FTD	NA	70	1 bulbar
Liu et al. (12)	China	434 SALS	2 (0.46%)	SALS	NA	53, 55	1 bulbar + 1 limb
		50 FALS	0	–	–	–	–
Ma et al. (13)	China	Case report	NA	FTD	NA	65	Personality change
Our case	China	Case report	NA	ALS-PSP	Rapidly progressive; atypical	73	Extrapyramidal symptoms

ALS, amyotrophic lateral sclerosis; FALS, familial ALS; SALS, sporadic ALS; hIBM, inclusion body myopathies; FTD, frontotemporal dementia; ALS-FTD, amyotrophic lateral sclerosis with co-morbid frontotemporal dementia; PSP, progressive supranuclear palsy; ALS-PSP, amyotrophic lateral sclerosis with co-morbid PSP-like symptoms; NA, no information available. Multicenter^*^, the 694 patients with FALS came from multiple countries including the UK (193), Italy (138), Spain (33), Germany (25), Ireland (17), the Netherlands (9), Belgium (3), New Zealand (1), the United States (266), and Canada (9); ALS^*^, it is only mentioned as ALS phenotype, while not definitely classified as SALS or FALS in the article.

## Discussion and conclusion

We report a case who initially presented with PSP-like symptoms, combined with prominent pyramidal features and cognitive impairment, and eventually developed extensive lower motor neuron damage. A missense mutation c.119A > G (p.D40G) of the *ANXA11* gene was identified using whole-exome sequencing, which confirmed the diagnosis of ALS. This is the first reported case of *ANXA11*-related ALS (*ANXA11*-ALS) presented with ALS-PSP phenotype, and the patients with *ANXA11* p.D40G-relevant ALS reported previously all presented with classical ALS phenotype without cognitive impairment.

*ANXA11* mutations were first reported to be associated with ALS in 2017 (5). Thus far, there are 68 ALS-relevant cases with *ANXA11* mutations including our case and 29 *ANXA11* variants have been identified. Four variants are considered to be pathogenic or likely pathogenic, including p.D40Y, p.D40G, p.G38R, and p.A58_Q187del (7–9, 11). Most *ANXA11*-related ALS cases presented as typical ALS phenotype, while there was one Chinese patient with ALS with the *ANXA11* p.P36R variant reported by Zhang et al. presenting as ALS-FTD phenotype (11), and some patients with the *ANXA11* p.D40Y variant reported in Brazilian families presenting as inclusion body myopathy (hIBM) apart from ALS (7). This indicates the phenotypic heterogeneity with *ANXA11* mutations even within the same pedigree. Our case is the first reported case with ALS-PSP phenotype, and except for our patient, cognitive impairment has not been reported in previous patients with ANXA11 p.D40G-relevant ALS.

Functional data showed that p.D40G is located in proximity to the calcyclin-binding region, and the variant could result in abnormal binding of calcyclin (5). Transfected human embryonic kidney cells expressing *ANXA11* with the p.D40G mutation showed altered binding to calcyclin (5). Liao et al. (14) proved that p.D40G mutation could reduce the stability of the *ANXA11* protein. Smith et al. (5) found that *ANXA11*-positive protein aggregates were abundant in spinal cord motor neurons and hippocampal neuronal axons in a patient with ALS carrying the p.D40G mutation.

Although there is no positive family history of this patient, we know that all ALS-associated genes and many other genes associated with related conditions show age-dependent penetrance, with the risk of disease manifestation increasing with age and some of the implicated genes are incompletely penetrant (15, 16), family size is becoming smaller and smaller, family members may die due to other causes before the onset of the disease, and these are all the reasons that the patients may show negative family history. In fact, about 10% of patients with SALS have gene mutations, and first-degree relatives of patients with SALS are at an 8-fold higher risk of developing the disease (1, 17). Thus, whether to take genetic testing only depending on the family history will miss some genetic variations in the clinical practice, especially with smaller families becoming the norm. In fact, the patient's only daughter also carries the same mutation as her father but does not show any clinical symptoms. She is 48 years old, so maybe her risk to have the disease will increase with aging but still has the opportunity to remain normal as Hardiman et al. (15) have indicated that familial forms of ALS are often characterized by < 50% penetrance. A meta-analysis aiming to determine the genetic features of ALS in the Chinese population showed that, in Chinese SALS, the highest mutation frequency was identified in the *SOD1* gene (1.6%), followed by *FUS* (1.3%), *SQSTM1* (1.0%), *OPTN* (0.9%), and *CCNF* (0.8%) (1). While in 2018, Zhang et al. (11) recruited 353 Chinese patients with SALS to investigate the genetic contribution of *ANXA11* by Sanger Sequencing and concluded that *ANXA11* mutation accounted for a mutant frequency of 2.3% in SALS. It seems that the *ANXA11* mutation is the leading gene in Chinese SALS. In the same year, Tsai et al. (10) screened a cohort of 244 Taiwanese patients with SALS using the same method and found eight missense variants in the *ANXA11* gene but only one variant was absent from population databases. Combined with these two studies, the mutant frequency of *ANXA11* is about 1.4%. According to these data, *SOD1* may be still the most common gene mutation in Chinese SALS, and *ANXA11* followed as the second.

The classic phenotype of ALS is characterized by the degeneration of upper and lower motor neurons, while behavioral and cognitive impairments are also common symptoms. According to previous studies, approximately 50% of cases show various degrees of cognitive impairment, from mild to FTD (18, 19). Generally, *C9orf72, TARDBP*, and *TBK1* variations often combine with cognitive impairment, while *SOD1* is known as “Pure” ALS genes (20–23). Thus far, the clinical phenotype spectrum of *ANXA11* is unclear. According to the previously reported cases, the *ANXA11* variant can present with classical SALS, FALS, classic motor symptoms with co-morbid FTD, and even ALS, hIBM, and ALS plus hIBM in one pedigree simultaneously. Our patient carrying *ANXA11* mutation presented with ALS-PSP phenotype, and this is the first reported case of this phenotype. Many genes associated with ALS are pleiotropic. For example, the mutation in valosin-containing protein (*VCP*) has been detected in family pedigrees with heterogeneous phenotypes such as ALS, FTD, hIBM, and Paget disease of bone (24). Hence, in 2013, Benatar et al. (25) proposed the concept of multisystem proteinopathy (MSP), which is an inherited pleiotropic degenerative disorder that can affect muscle, bone, and the nervous system. In 2015, Taylor et al. (26) proposed an operational definition of MSP, which is a combination of two or more phenotypes of hIBM, Paget disease of bone, ALS, or FTD. According to these criteria, Leoni et al. (7) proposed that *ANXA11* should be considered as a gene associated with a novel type of MSP (MSP type 6), rather than just an ALS-related gene.

Our patient presented with prominent PSP-like symptoms initially, including repeated falls and abnormal eye movement, which is rare in ALS. In fact, there were a few studies that have addressed the patients with ALS accompanying extrapyramidal symptoms including both hyperkinetic and hypokinetic movement disorders (27–29). In 2019, Calvo et al. (29) recruited 101 patients with ALS and identified 31 patients (30.7%, 31/101) with the co-morbid parkinsonian disorder (ALS-PK), who showed bradykinesia (100%), axial symptoms (100%), rigidity (89.2%), tremor (57.1%), and cognitive impairment (35.7%). They detected four mutations in four of the 31 patients with ALS-PK, including *C9orf72* (3.2%, 1/31), *TARDBP* (3.2%, 1/31), *LRRK2* (3.2%, 1/31), and *PARK2* (3.2%, 1/31). However, *ANXA11*-ALS who showed an atypical ALS with PSP-like symptoms has not been previously reported. In 2012, D'Ascenzo et al. (30) enrolled 16 patients with ALS with predominant upper motor neuron involvement and extrapyramidal-like features and found eight of them (50%, 8/16) showed a slight to a severe reduction in striatal dopamine transporter-positron emission tomography uptake. Unfortunately, due to poor physical condition, our patient could not complete the dopamine transporter-positron emission tomography. In 2019, a cohort of 97 autopsied cases of sporadic ALS was examined by Ito et al. (27). They identified 11 cases (11.3%, 11/97) who showed pallidonigroluysian degeneration (PNLD), and two patients with PNLD (18.2%, 2/11) developed extrapyramidal signs as the initial symptoms, while extrapyramidal signs were not observed in the remaining 86 cases without PNLD. Thus, they thought that PNLD accounted for the early development of extrapyramidal signs. By taking levodopa, our patient could improve his muscle rigidity to some extent, and thus, we speculated that the pallidonigroluysian system of this patient might be affected.

We report a case of sporadic ALS with PSP-like symptoms. Genetic testing confirmed the *ANXA11* p.D40G variant. Through the literature review, we found 68 ALS-relevant patients with *ANXA11* mutations, which presented with typical ALS, hIBM, FTD, or a combination of these phenotypes. Our ALS-PSP phenotype is first reported to be associated with *ANXA11*. According to the previous reports, 29 heterozygous nonsynonymous *ANXA11* variants were identified, and p.D40Y, p.D40G, p.G38R, and p.A58_Q187del are identified as pathogenic or likely pathogenic. Most patients with ALS with the *ANXA11* p.D40G variant presented with a classical ALS phenotype without cognitive impairment except *for* our patient. Thus far, due to the limited number of cases, the genotype–phenotype correlation in *ANXA11*-ALS is not clear. As we learn more about *ANXA11* variation, we may have a deeper understanding of its variety and phenotype in the future.

## Data availability statement

The datasets presented in this study can be found in online repositories. The names of the repository/repositories and accession number(s) can be found in the article/Supplementary material.

## Ethics statement

The studies involving human participants were reviewed and approved by the Ethical Committee of Xuanwu Hospital of Capital Medical University. Written informed consent was obtained from the patient's only daughter for the publication of any potentially identifiable images or data included in this article.

## Author contributions

XZ analyzed and interpreted the data and wrote the manuscript. JG, CC, ZZ, and PC analyzed and interpreted the data. JM designed and conceptualized the study, interpreted the data, and revised the manuscript. All authors contributed to the article and approved the submitted version.

## Supplementary material

Supplementary Figure S1 Geographic distribution of 68 affected subjects with *ANXA11* mutations. Multicenter^*^, nine patients with *ANXA 11* mutations from 694 patients with FALS coming from multiple countries including the UK (193), Italy (138), Spain (33), Germany (25), Ireland (17), the Netherlands (9), Belgium (3), New Zealand (1), the United States (266), and Canada (9).

Supplementary Figure S2 The phenotypes of 68 cases with *ANXA11* mutations. ALS, amyotrophic lateral sclerosis; SALS, sporadic ALS; FALS, familial ALS; hIBM, inclusion body myopathies; ALS^*^, it is only mentioned as ALS phenotype, while not definitely classified as SALS or FALS in the article; hIBM+ALS, hIBM with co-morbid ALS; FTD, frontotemporal dementia; ALS-FTD, ALS with co-morbid FTD; ALS-PSP, ALS with co-morbid PSP-like symptoms.

Supplementary Table S1 Clinical phenotype of the patients with *ANXA11* p.D40G-relevant ALS.
