# Supplementary material for: An atypical ALS with PSP-like symptoms caused by ANXA11 p.D40G mutation: A case report and literature review
Source: Front Neurol. 2023 Feb 16;14:1086264. doi: 10.3389/fneur.2023.1086264 (PMC9978770; doi:10.3389/fneur.2023.1086264)
Supplement: Supplementary Figure S1 — Geographic distribution of 68 affected subjects with ANXA11 mutations. Multicenter*, nine patients with ANXA 11 mutations from 694 patients with FALS coming from multiple countries including the UK (193), Italy (138), Spain (33), Germany (25), Ireland (17), the Netherlands (9), Belgium (3), New Zealand (1), the United States (266), and Canada (9). [file Data_Sheet_1.docx]

Supplementary Material


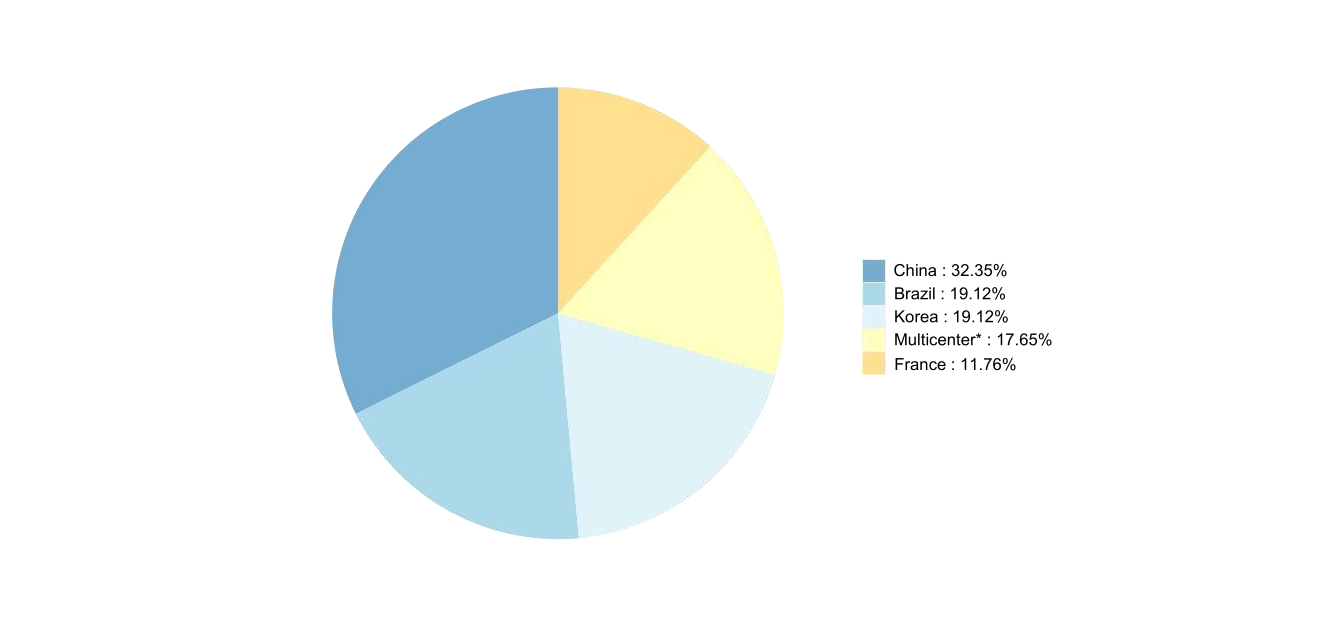


**Figure S1.** Geographic distribution of sixty-eight affected subjects with *ANXA11* mutations.

**Abbreviation:** Multicenter*, nine patients with *ANXA 11* mutations from 694 FALS patients coming from multiple countries including U.K. (193), Italy (138), Spain (33), Germany (25), Ireland (17), Netherlands (9), Belgium (3), New Zealand (1), the United States (266) and Canada (9).


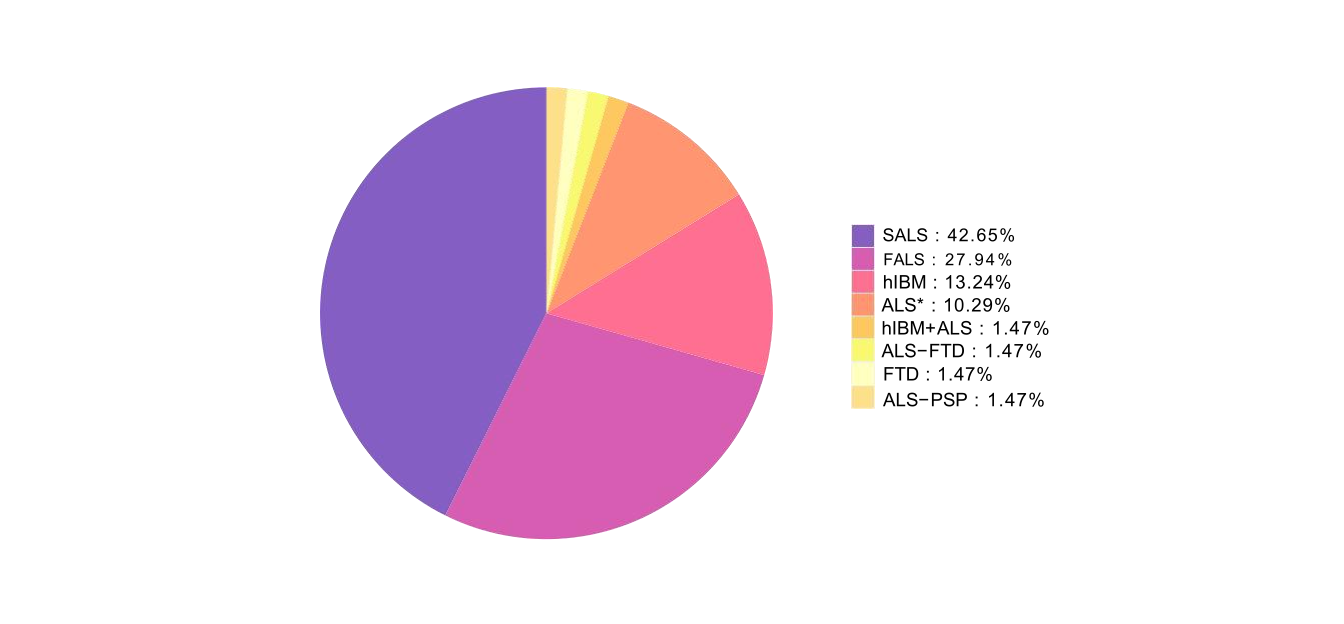


**Figure S2.** The phenotypes of sixty-eight cases with *ANXA11* mutations.

**Abbreviation:** ALS, amyotrophic lateral sclerosis; SALS, sporadic ALS; FALS, familial ALS; hIBM, inclusion body myopathies; ALS*, it's only mentioned as ALS phenotype, while not definitely classified as SALS or FALS in the article; hIBM+ALS, hIBM with co-morbid ALS; FTD, frontotemporal dementia; ALS-FTD, ALS with co-morbid FTD; ALS-PSP, ALS with co-morbid PSP-like symptoms.

**Table S1.** Clinical phenotype of the *ANXA11* p.D40G relevant ALS patients.

| **References** | **Source** | **Phenotype** | **Gender** | **Age of onset** | **Initial symptoms** | **With or without cognitive impairment (Y/N)** |
| --- | --- | --- | --- | --- | --- | --- |
| Smith et al. (5) | Multicenter* | FALS | F | 74 | bulbar | N |
|  |  | FALS | F | 70 | limb | N |
|  |  | FALS | F | 55 | bulbar | N |
|  |  | FALS | F | 72 | bulbar | N |
|  |  | FALS | M | 83 | bulbar | N |
|  |  | SALS | M | 72 | bulbar | N |
| Nahm et al. (9) | Korea | SALS | M | 68 | bulbar | NA |
| Zhang et al. (11) | China | SALS | F | 59 | limb | N |
| Our case | China | ALS-PSP | M | 73 | extrapyramidal symptoms | Y |

**Abbreviation:** ALS, amyotrophic lateral sclerosis; FALS, familial ALS; SALS, sporadic ALS; PSP, progressive supranuclear palsy; ALS-PSP, ALS with co-morbid PSP-like symptoms; F, female; M, male; Y, with cognitive impairment; N, without cognitive impairment; NA, no information available. Multicenter*, the 694 FALS patients came from multiple countries including U.K. (193), Italy (138), Spain (33), Germany (25), Ireland (17), Netherlands (9), Belgium (3), New Zealand (1), the United States (266) and Canada (9).
